# Individualized patient decision-aid for immunosuppressive drugs in women with lupus nephritis: study protocol of a randomized, controlled trial

**DOI:** 10.1186/s12891-017-1408-5

**Published:** 2017-01-31

**Authors:** Jasvinder A. Singh, Nipam Shah, Candace Green

**Affiliations:** 1 Rheumatology Section, Medicine Service, VA Medical Center, Birmingham, AL USA; 2 Division of Rheumatology, Department of Medicine, University of Alabama at Birmingham, Birmingham, AL USA; 3 Division of Epidemiology at the School of Public Health, University of Alabama at Birmingham, Birmingham, AL USA

**Keywords:** Lupus, Lupus nephritis, Minorities, Women, Decision-aid, Randomized trial, Randomized controlled trial

## Abstract

**Background:**

Systemic Lupus erythematosus (SLE), also commonly referred to as lupus, is a rare, but sometimes, fatal disease, that primarily affects young women. Lupus nephritis, a common manifestation of lupus, is more common and more devastating in patients of minority race/ethnicity. Patients have negative views of immunosuppressive drugs for lupus nephritis due to a concern about side effects and under-appreciation of its benefit. We designed a study to assess the effectiveness of individualized, computerized patient decision-aid for immunosuppressive drugs for lupus nephritis compared to a standard pamphlet for patient decision-making.

**Methods:**

Adult women with lupus nephritis, with a current lupus nephritis flare or at risk of a future lupus nephritis flare will be randomized to individualized, computerized patient decision-aid for immunosuppressive drugs vs. standard pamphlet with information about lupus and its treatment including immunosuppressive drugs and outcomes. Patients will complete outcome assessments immediately after the intervention has been administered. Patients will be followed at 3-months with a brief survey, either in person or on the phone, and at 6-months with medical record review for exploratory outcomes. Co-primary outcomes are decisional conflict and informed choice regarding immunosuppressive drugs (combines values, knowledge and choice). Secondary outcomes include: (1) assessment of patient-physician communication by assessing audio-taped physician-patient communication after intervention administration; (2) concordance between patient’s desired and actual role in immunosuppressive drugs decision-making using the control preference scale (CPS); and (3) patient perception of physician interaction using the interpersonal process of care- short form (IPC-SF).

**Discussion:**

This is one of the first studies to evaluate the effectiveness of an educational intervention targeting minorities with lupus nephritis. This patient-centered lupus nephritis decision-aid will be available in the public domain in English and Spanish.

**Trial registration:**

ClinicalTrials.gov Identifier: NCT02319525; registered on November 5, 2014.

## Background

In the US, 161,000 individuals have definite SLE [1], making it a rare disease with a prevalence of 0.05%. However, lupus nephritis accounts for 2% of all end-stage renal disease in the U.S. [2], a proof of its devastating effect on kidneys, making it a serious disease. Compared to Whites, African-Americans and Hispanics not only have a higher incidence of lupus and higher associated mortality [3–6], but also a higher prevalence of lupus nephritis and worse renal outcome [6–14]. Thus, more research is needed in minorities with lupus nephritis. Research in rare diseases is an area of focus of patient-centered outcomes research institute (PCORI) funding.

Many racial/ethnic minority patients do not receive quality health care, due in part to lower health literacy and numeracy [15], and poorer physician-patient communication [16–19]. Compared to Caucasians, African-American women with lupus have a lower likelihood of accepting immunosuppressive drugs such as cyclophosphamide [20, 21]. Immunosuppressive drugs in combination with corticosteroids are the standard of care for induction treatment of lupus nephritis treatment rather than either medication alone; immunosuppressive drugs are also important in the maintenance phase of the treatment of lupus nephritis [22, 23].

There are many unanswered questions regarding the patient perception of immunosuppressive drugs in patients with lupus, especially in minority patients with lupus. To our knowledge, there is little or no evidence about whether interventions to change patient knowledge or perception regarding immunosuppressive drugs in lupus patients can improve decision-making regarding these treatments. Compared to Caucasians, studies show that African-Americans had lower numeracy, which explained poor chronic disease control in diabetes [24], and HIV medication regimen [25]. A decision-aid can provide information tailored to patients with low health literacy and numeracy. Therefore, the objective of this study is to compare the efficacy of the usual education materials to individualized computerized decision-aid (guide) to reduce the decision conflict regarding treatment decisions for immunosuppressive therapies in patients with lupus nephritis. Our long-term objective is to improve outcomes in patients with lupus.

## Methods

### Study overview and hypothesis

Some patients with lupus have negative views of immunosuppressive drugs (cyclophosphamide, mycophenolate mofetil, azathioprine, calcineurin inhibitors such as cyclosporine and tacrolimus etc.) and may reject consideration of these drugs for treatment of their lupus [26–29]. The rejection of immunosuppressive drugs may be related not on their values, but also to limited knowledge of efficacy of these drugs coupled with a fear of adverse events. As a result, many patients choose to use only corticosteroids rather than a combination of immunosuppressive drugs and corticosteroids, for the treatment of their active lupus nephritis. The combination is not only more effective than corticosteroids alone for lupus nephritis, but in many cases, may also be associated with similar or lower risk of adverse events [22, 23].

This study will test the effectiveness of a decision-aid regarding immunosuppressive drugs in patients with lupus nephritis, with a focus on minority race/ethnicity. It is a comparative effectiveness research (CER) study. The main study aim is to assess the effectiveness of an individualized computerized patient decision-aid focused on immunosuppressive drugs for lupus nephritis. Our hypotheses are that in patients with lupus nephritis, compared to usual care (educational pamphlet), individualized computerized decision-aid will be more effective, as indicated by a greater reduction in decisional conflict scores (*Hypothesis 1*) and a higher rate of informed choice favoring immunosuppressive drugs (concordance between values, knowledge and choice of immunosuppressive drugs; *Hypothesis 2*).

The study protocol is registered at the clinicaltrials.gov website, NCT02319525. All study visits, including screening, baseline visit and 3-month follow-up assessments will be done during patient’s regular scheduled clinic visits/appointments, with few exceptions, if any. This was done in order to keep the patient burden for study participation low and to encourage patients with lower socio-economic status and/or difficulty in transportation to participate in this research study focused at disadvantaged patients with limited resources.

### Study design

This is a multicenter, parallel two-arm, prospective randomized trial comparing an individualized computerized decision-aid tool to an educational pamphlet (usual care). Patients with lupus nephritis attending clinics at the 4 recruiting sites will be recruited over a 2-year period starting January 2015. Local Institutional Review Board at each participating site, including the University of Alabama at Birmingham (UAB), approved the study procedures. This study was funded by a grant from the Patient Centered Outcomes Research Institute (PCORI).

### Study population

We will oversample African American and Hispanic groups due to study’s focus on minorities, but other racial groups will also be recruited in the study. Patients with lupus nephritis will be enrolled from participating clinics at UAB, University of California at San Francisco, Baylor College of Medicine and Ohio State University. These sites were chosen based on high numbers of minority lupus patients, and a dedicated lupus clinic/service at each site. We will recruit two groups of patients, including patients with: (1) newly diagnosed active lupus nephritis needing immunosuppressive therapy or a lupus nephritis flare despite current immunosuppressive therapy requiring a change in immunosuppressive drug/therapy (current lupus nephritis flare scenario); or (2) lupus nephritis with prior experience and/or discussion regarding immunosuppressive therapy for a past lupus nephritis flare, who are at the risk of a future lupus nephritis flare (future lupus nephritis flare scenario). Decisional conflict and informed choice were co-primary outcomes and secondary outcomes included patient decision-making and patient-physician communication.

### Study intervention

The study intervention is an educational behavioral intervention targeting patient knowledge and opinions about immunosuppressive drugs. Active intervention includes an individualized, computerized decision-aid, developed specifically for this study, with a focus on minority women. The control intervention is a pamphlet with information about lupus and its treatment.

The decision-aid was developed in multiple steps incorporating the following elements: (1) comparative effectiveness data (benefits and common and uncommon harms) on immunosuppressive drugs with systematic reviews, meta-analysis and network meta-analysis (NMA) incorporating evidence from direct comparison studies and indirect comparisons [30–32]; (2) qualitative assessment of patient barriers an facilitators to decision-making regarding immunosuppressive medications [33, 34]; and (3) iterative testing and modification of the decision-aid by piloting in English- and Spanish speaking lupus patients at multiple study sites to ensure that words, phrases, messages and images used were acceptable and could be easily understood considering health literacy, numeracy and graphical literacy of the target population.

The decision-aid tool has been specifically designed in English and Spanish to assist patients with lupus nephritis make decisions regarding immunosuppressive drugs. The decision-aid will provide individualized information about immunosuppressive drugs specific to the each decision time point (induction vs. maintenance). We developed this tool for four probable scenarios based on the most common scenarios for a choice of immunosuppressive drugs (details in the section below). The decision-aid for each scenario will provide information about lupus in general, how lupus affects kidneys, benefits and side effects of using corticosteroids, general information (medication formulation, route of administration, costs of medication, dosage) and comparative risks and benefits about two immunosuppressive being compared, a final summary of the information provided and reference to other patient support groups. Patients are prompted to write down questions for their physician about these choices. The computerized decision-aid is programmed such that once the scenario is chosen by the coordinator per the guidance of the referring physician based on the most likely two choices (or a provider in the future), the decision-aid only shows that comparison.

Usual care or control group will receive a paper copy of the American College of Rheumatology pamphlet with information on lupus nephritis which is freely available online on the American College of Rheumatology website [35]. The pamphlet provides information about lupus in general, causes of lupus, how lupus is diagnosed and treated, broader health impact of lupus and living with lupus.

### Study procedures

#### Screening

**Fig. 1:**
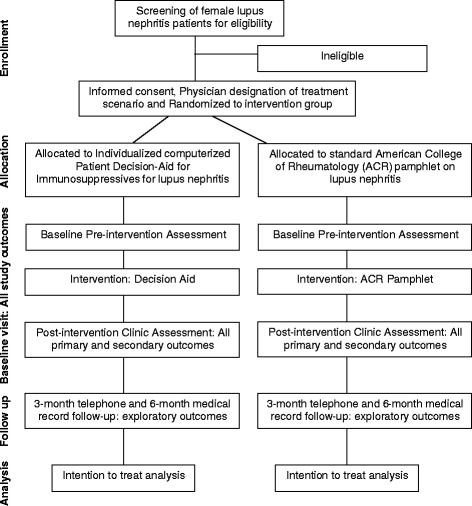
CONSORT diagram showing each stage of the randomized trial

We will use International Classification of Diseases, ninth revision, common modification (ICD-9-CM) code for lupus, 710.0, to generate a list showing hospital clinic appointments of lupus nephritis patients, their gender and race/ethnicity each month. We will screen this list to identify potentially eligible individuals weekly, and discuss each patient’s potential eligibility for the study with the health care provider for their lupus care, ahead of their visit (Table 1, study eligibility criteria). We will also screen the lists of patients scheduled for lupus clinics at each institution, since each participating institution has a weekly lupus clinic. Male patients and patients with lupus but no evidence of lupus nephritis will be excluded. In addition, this study will be advertised in the rheumatology and nephrology clinics, to rheumatology and nephrology fellows and attending physicians, who frequently follow patients with lupus nephritis, and to renal pathologists at each institution. The study coordinator will examine clinic notes for eligibility criteria for patients referred to us by physicians, fellows or renal pathologists. We will find out the scheduled clinic appointments for these potentially eligible patients, and inform their treating physician about potential patient study enrollment on the day of the clinic visit. To ensure timely patient enrollment, we will have regular meetings with rheumatology and nephrology attending physicians and fellows who primarily provide care to lupus patients, as well as send periodic emails to all providers. A brief overview of the study flow is provided in the CONSORT diagram (Fig. 1).

**Table 1:** Study eligibility criteria

Inclusion criteria
1. Females with lupus nephritis 2. Currently having a flare of lupus nephritis according to expert rheumatologist and considering change or initiation of an immunosuppressive medication for lupus nephritis (current flare) or had had flare of lupus nephritis in the past and have had experience or discussion of immunosuppressive medication for lupus nephritis who were at risk for a future flare (future flare) 3. Age 18 years or older 4. All racial/ethnic groups
Exclusion Criteria
1. Male patients with lupus nephritis 2. Patients with lupus but no nephritis 3. Patients having kidney flare but medication change is not considered 4. Patients with end stage renal disease on dialysis 5. Patients with a renal transplant or who are candidate for a renal transplant

#### Baseline visit: procedures at initial patient visit

##### Physician Designation of the treatment scenario and Informed Consent

Before obtaining informed consent from a study subject, the physician will be notified of the potential recruitment and asked to assign a scenario (medication group; details in the relevant section below) and whether the patient having a flare of lupus nephritis currently (current lupus nephritis flare scenario) or is at risk of a flare in the future (future lupus nephritis flare scenario). Study coordinator will then obtain a written informed consent.

##### Randomization

After informed consent is obtained, subjects will be randomized using the Research Electronic Data Capture (REDCap) electronic data capture tool [36]. Once the randomization form is completed and saved, a unique participant identification number will be generated. Randomization will be stratified by study site and language (English vs. Spanish). Study coordinators will enter patients’ name, date of birth, gender, and recruitment site in the REDCap randomization form. A computer-generated algorithm will randomize assign study subjects to either decision-aid or pamphlet intervention groups in a 1:1 ratio. We will use a separate unique prefix for each site added to each patient identification number in order to identify subjects by study site.

##### Pre-intervention assessment

After each subject is randomized, the study coordinator will administer assessments of health literacy using Rapid Estimate of Adult Literacy in Medicine-Short Form (REALM-SF) [37] and Short Assessment of Health Literacy (SAHL) [38] and graphical and numeric literacy to them. Study subjects will also respond to questions assessing knowledge and patient values about the immunosuppressive drugs for lupus nephritis as treatment options for them (current flare or future flare scenarios), and choose what treatment they prefer now for their current (or future) lupus nephritis flare. Pre-intervention assessments will evaluate primary outcomes such as decisional conflict scale [39] and patient’s choice of the immunosuppressive drug based on knowledge and values related to taking immunosuppressive drugs (informed choice; details in the section on outcome measures below) [40, 41]. All these assessments will be completed before administering the study intervention.

##### Intervention and scenarios

Patients will read the educational material, i.e., decision-aid or the pamphlet, based on the group they are randomized to, i.e., computerized decision-aid or the pamphlet. This section provides details of the intervention, and each intervention arm.

Decision-aid: In addition to the information on lupus nephritis, the decision-aid will provide information on four scenarios of choice between immunosuppressive drugs. The scenarios were chosen by the patient’s lupus care provider based on most likely choices for patient’s treatment, based on either their current situation (current flare) or anticipated future lupus nephritis flare (future flare). Details are given below:

Scenario A (Treatment induction in immunosuppressive-naive): This will be applicable to patients with newly diagnosed lupus nephritis or having history of lupus nephritis with deteriorating renal function, who are currently not on any immunosuppressive medication (current flare) or with stable renal function currently not requiring immunosuppressive drug, but at the risk of future flare requiring induction therapy (future flare). The decision-aid tool for this scenario will provide information about two medication choices: cyclophosphamide (cytoxan) vs. mycophenolate mofetil (cellcept).

Scenario B (Treatment maintenance in azathioprine failure): This will usually be applicable to patients with lupus nephritis who are currently on azathioprine (Imuran) and have deteriorating renal function (azathioprine failure; current flare) or stable renal function on current azathioprine, but at the risk of future flare requiring change in maintenance therapy (future flare). The decision-aid tool for this scenario will provide information about two medication choices: cyclophosphamide (cytoxan) vs. mycophenolate mofetil (cellcept).

Scenario C (Treatment maintenance in azathioprine and mycophenolate mofetil failure): This will usually be applicable to patients with lupus nephritis who have failed azathioprine (Imuran) and mycophenolate mofetil (cellcept) therapy and thus have signs of deteriorating renal function (current flare) or stable renal function on current immunosuppressive maintenance therapy, but at the risk of future flare requiring change in maintenance therapy (future flare). The decision-aid tool for this scenario will provide information about two medication choices: cyclophosphamide (cytoxan) vs. calcineurin inhibitors (cyclosporine, tacrolimus etc.).

Scenario D (Treatment maintenance in mycophenolate mofetil failure): This will usually be applicable to patients with lupus nephritis who are currently on mycophenolate mofetil (cellcept) and have deteriorating renal function (cellcept failure; current flare) or stable renal function on current mycophenolate mofetil maintenance therapy, but at the risk of future flare requiring change in maintenance therapy (future flare). The decision-aid tool for this scenario will provide information about two medication choices: cyclophosphamide (cytoxan) and azathioprine (Imuran).

Pamphlet: This is a standard material available online from the American College of Rheumatology (ACR) (3). It will be provided as a paper pamphlet to the patient to read and review. The pamphlet provides information about lupus, symptoms of lupus, diagnosis of lupus, laboratory test for lupus and tips to control lupus in lay language. It also provides web links to various support groups for lupus.

##### Post-intervention assessment, including the Audio taping of the physician-patient communication

After the administration of the intervention, patients will have a discussion with physicians about immunosuppressive drugs (only current flare patients) and all study subjects will complete the post-intervention assessment questionnaire. This questionnaire will measure co-primary outcomes (decision conflict and informed choice) as in pre-intervention assessments. In addition to this, the post-intervention questionnaire will measure two secondary outcomes, i.e., the control preference scale (CPS) [42–44] and interpersonal process of care- short form (IPC-SF) [45]. Patient-physician conversation will be audio taped for all patients with current lupus nephritis flares (current flare patients), but not future flare patients, and assessed as a secondary outcome. The audio taped conversation constitutes one of the three secondary outcomes.

#### 3-month phone vs. clinic follow-up assessment

At 3 months, study subjects will respond to the IPC questionnaire. Subjects will respond to this questionnaire via phone or during a routine clinic visit, if it coincides with study follow-up date. When subjects are not reachable via phone and not seen in clinic, the study coordinator will mail the follow up questionnaire.

#### 6-month medical record follow-up assessment

The site study coordinators will extract laboratory, medication and other clinical data on exploratory outcomes at 6 months using Electronic Health Record (EHR) at each study site for patients currently having lupus nephritis flare (current flare scenario), except missed/cancelled outpatient clinic visits which will be captured for all patients. The exploratory outcomes include cumulative glucocorticoid dose, immunosuppressive medication adherence and persistence, serum creatinine and spot protein/creatinine ratios, and the number of missed/cancelled clinic appointments. We anticipate that due to the variation of the frequency and type of regular laboratory assessments of lupus nephritis across sites, there will be significant amount of missing data. We also anticipate that available laboratory data is likely to be collected at time points different from the 6-month study follow-up time-point, since these are being done as part of clinical care not as part of trial protocol, making it heterogeneous and sometimes not usable. We will attempt to analyze these data as rigorously as possible. We considered obtaining these data for our study, but patient stakeholders advised us to not add additional assessments or additional clinic or laboratory visits to capture outcomes not directly related to the study intervention.

### Outcome measures

#### Co-primary outcome measures

Decisional Conflict Scale (DCS), low-literacy version: Decision conflict scale is a patient self-administered, validated measure of decisional conflict, most commonly used as the primary outcome in RCTs of decision-aid [46, 47]. We will assess the change in decisional conflict related to immunosuppressive drugs after the administration of the intervention at the baseline visit and compare the change from baseline  between the groups (Table 2). The low literacy version consists of 10 items with 3 response categories (yes, unsure, no) with overall score of 0 (no decisional conflict) to 100 (extreme decisional conflict) [48]. Responses are given the following score values: yes = 0; unsure = 2; no = 4. Ten items are summed and multiplied by 2.5 to provide a score ranging 0-100. It is available in English and Spanish versions. Decisional conflict represents a state of uncertainty about a choice or course of action and is more likely in situations involving high-stakes choices with important potential gains and losses, value tradeoffs in selecting a choice or a course of action (vs. the alternative) or uncertain outcomes. The DCS scale also has 4 subscales: (1) Uncertainty subscore: Scores range from 0 (feels extremely certain about best choice) to 100 (feels extremely uncertain about best choice); (2) Informed subscore: Scores range from 0 (feels extremely informed) to 100 (feels extremely uninformed); (3) Values Clarity subscore: Scores range from 0 (feels extremely clear about personal values for benefits and risks/side effects) to 100 (feels extremely unclear about personal values for benefits and risks/side effects); and (4) Support subscore: Scores range from 0 (feels extremely supported in decision making) to 100 (feels extremely unsupported in decision making). DCS subscale scores will be explored, if data are available for comparisons.

**Table 2:** Study Outcomes and Outcome measures

Co-Primary outcomes/ Time of assessment	Description
Change in Decisional Conflict Scale (DCS) score [39, 46–48, 64]/Baseline post-intervention	10-item patient-reported scale for lower literacy populations with 3-level response categories
Informed choice [40, 41]/Baseline post-intervention	Concordance between patient’s values related to taking immunosuppressive drugs and patients’ decision to start immunosuppressive drugs in those with adequate knowledge about immunosuppressive drugs
Secondary outcomes	
Control Preferences Scale [51]/Baseline post-intervention	2-item patient-reported assessment of whether the decision-making concordant with patient's preference about their role
Interpersonal Processes of Care [45]/Baseline post-intervention	18-item patient-reported multidimensional measure of physician-patient communication
Audiotaped Patient-physician discussion [56]/Baseline post-intervention	Active Patient Participation Coding Scheme decoding speech acts indicating patient participation and whether physician communication was patient-centered

##### Informed choice

We will assess informed choice by using a validated multidimensional model of informed choice [40, 41] that individually assesses and then combines three constructs: values regarding immunosuppressive drugs, knowledge about immunosuppressive drugs, and treatment choices. We will assess informed choice after patient has viewed the decision-aid or lupus pamphlet at the baseline study visit before any treatment decision-making. *Values* will be assessed with a list consisting of patients’ views regarding immunosuppressive drugs as treatment option and their side effects generated by us based on patient concerns about regarding immunosuppressive drugs. The values statements consisted of both positive and negative values about immunosuppressive drugs, mixed in a random order, with responses ranging from strongly disagree to strongly agree. We will score positive and negative value statements with appropriate signs (+ or -) and aggregate into a total score. A higher total score will indicate more positive values regarding using immunosuppressive drugs and a median score (or an overall more positive vs. more negative values) will be used to classify values as positive vs. negative regarding using immunosuppressive drugs. *Knowledge* regarding immunosuppressive drugs for lupus nephritis were assessed using 20 questions and considered “adequate knowledge” if patients answer  at least 75% of questions correctly. *Choice* will be assessed based on response to a single item on a nominal scale with anchors of start vs. don’t start immunosuppressives and “uncertain” in the middle and 15 circles, asking patient’s choice in response to a question “if your doctor asked you right now to make a choice about immunosuppressives, please show where you would be on the scale below by choosing a circle below”. Informed choice refers to a choice that is based on accurate knowledge and is concordant with one’s values. A higher proportion indicates more patients with informed choice regarding immunosuppressive drugs. 

#### Secondary outcome measures

##### Control preferences Scale

This validated measure will assess patient participation in decision-making for patients with lupus nephritis flare (current flare scenario patients only). This assesses how much decision-making control patients would like to have versus actually experienced by each patient. It determines the correlation between patient satisfaction with care and discriminates between those who feel involved in the decision versus not [42–44]. There are 5 responses for 5 control options: active, active shared, collaborative, passive shared and passive, which will be categorized as active (combining active and active shared), collaborative and passive (combining passive and passive shared), as previously [49–51]. We will examine for concordance between desired and actual roles played by patients with lupus nephritis flare (current flare scenario). A higher concordance in roles (desired vs. actually played) indicates that more patients experienced the desired role in decision-making.

##### Patient physician communication and care processes

This will be assessed using the Interpersonal Processes of Care Short Form (IPC-SF), an 18-item validated patient-reported measure of patient-physician communication and care processes [45, 52–55]. IPC-SF is available in both English and Spanish versions.

##### Analysis of Audiotaped physician-patient interaction

This will be done recording the patient physician discussion about immunosuppressive drugs and using the Active Patient Participation Coding Scheme (APPC), a validated instrument to assess indicators and facilitators of patient participation [56], for patients with a current lupus nephritis flare (current flare scenario). Three types of speech acts will be coded as active patient participation, because of their potential to influence a doctor’s behavior as well as the content and structure of the consultation [57–60], namely, question-asking, assertive responses, and expressions of concern. In addition, physician communication will be coded using speech acts such as supportive talk and partnership building. Trained coders will transcribe and recognize active participation with ‘utterance’ as the unit of analysis (the oral analogue of a simple sentence, independent clause), which will be summed for each interaction to create a frequency of the degree of active participation.

#### Exploratory outcome measures

We will assess the following exploratory outcomes, where data are available. Most exploratory outcomes are applicable only to patients with current flare scenarios, except missed/cancelled appointments (current flare or future flare scenario). Outcomes include cumulative glucocorticoid dose, medication adherence and persistence with immunosuppressive drugs, renal function as assessed by spot protein/creatinine ratios, serum creatinine, 24-h proteinuria, proportion with complete or partial renal remission, number of missed or cancelled clinic appointments.

We will also assess the acceptability of study intervention using an acceptability survey [61], as in previous studies [62]. Patients will rate the quality and the quantity of the information presented in decision aids (4-point scale ranging from “excellent” to “poor”) and rate the appropriateness of amount of information (responder burden), presentation style and usefulness. Feasibility will be assessed by the amount of assistance required in navigating decision aids (none, a little, some, a lot) and with a self-administered satisfaction questionnaire [63]. Patients will rate satisfaction with decision aids on a 5-point Likert scale ranging from strongly agree to strongly disagree: Easy to use, practical, process did not take too long, easy to understand, did not mind spending extra time.

### Statistical analyses

#### Sample size calculation

Our study has an adequate power to assess the treatment effect for the co-primary outcomes, with an estimated enrollment of 200 patients. We anticipate that 100 African-American and 100 Hispanic/Caucasian females will lupus nephritis be enrolled in the trial. Allowing 10% loss to follow-up, this sample size will provide 80% power for co-primary outcomes. We will be able to detect a moderate effect size difference between group means on decisional conflict (range 0–100) using a two sample *t*-test and two tailed type I error rate of 0.05 (hypothesis 1) [48, 64] and a 15% absolute difference in the proportion of patients with informed choice using a one-sided type I error rate of 0.05 (hypothesis 2) [65].

#### Analysis of outcomes measures

We will compare demographic variables and baseline characteristics between decision-aid and pamphlet groups, including health, numeric and graphical literacy by comparing mean values or proportions as appropriate. Measures of variability (standard deviation) will be calculated. We will compare primary and secondary outcome measures using Student *t* test or analysis of variance or comparison of proportions. A two-sided *p*-value < 0.05 will be considered significant. We will stratify variables based on language (English/Spanish) and study sites and compare these variables using chi-square test or Fisher’s exact test. All statistical analyses will be carried out using SAS, v9.4 (Cary, NC). We will also use logistic or linear regression to assess other predictors of each of the co-primary outcomes, including the intervention.

### Study limitations

Our study has several limitations. We are assessing two co-primary outcomes. We chose these two co-primary outcomes, since they capture two complimentary aspects of decision-making, related to our intervention, an individualized decision-aid. If our intervention, an individualized decision-aid, is found to be effective, we will not know as to which individual component/message, led to the reduction in decision conflict or more informed choice. However, this is not a critical question to answer at this time, since as an educational intervention, our decision-aid is easy to implement. Since our decision-aid will be available in public domain, it would be available for any patient to use, and can be further modified to be contextually relevant. Another limitation is that while we have the decision aid and outcome instruments in English and Spanish, we do not have translations in other languages. This can be done in the future, and will make this decision-aid even easier to use for lupus patients who speak languages other than English and Spanish. In order to reduce the patient burden, we will not assess patient satisfaction and quality of life in this study. Therefore, the effect of this decision-aid on these domains will need to be examined in future studies.

## Discussion

This study is a 2-arm parallel group trial of a behavioral patient educational intervention on patient decision-making in women with lupus nephritis, with a focus on minority race/ethnicity. Decision-making for immunosuppressive medications is a challenge in the treatment of patients with lupus nephritis, especially in racial minorities, who have more severe disease and worse outcomes. Previous studies have shown that there are both knowledge gaps related to immunosuppressive drugs as well as patient views that do not favor the use of immunosuppressive medications in lupus nephritis [26–29]. To our knowledge, there are no trials of educational or behavioral interventions and few studies of interventions in minority patients with lupus nephritis, if any. Therefore, our study that investigates  if a patient-centered, individualized, easy to use intervention can reduce conflict in patient decisions and improve patient decision-making regarding immunosuppressive drugs for lupus nephritis, will fill an important knowledge gap.

## Conclusion

In conclusion, by its focus on the minority lupus patients who deal with a life-threatening complex illness with severe morbidity at a young age, our study results will pave the way for other studies of computerized patient decision-aid not only in lupus nephritis, but also for other similar complex illnesses (common and rare) with significant morbidity and mortality risk. This decision-aid will be in the public domain, available free of charge to everyone, for a free online download.

### Trial status

The study is ongoing. We are currently following patients enrolled at the four study sites.

## Abbreviations

ACR: American college of rheumatology
CER: Comparative effectiveness research
CPS: Control preferences scale
DCS: Decision conflict scale
ICD-9-CM: International classification of diseases, ninth revision, common modification
IPC: Interpersonal processes of care
RCT: Randomized controlled trial
REALM-SF: Rapid estimate of adult literacy in medicine-short form
SAHL: SDhort assessment of health literacy
SLE: Systemic lupus erythematosus
UAB: University of Alabama at Birmingham
